# The Role of Trifluoromethyl and Trifluoromethoxy Groups in Medicinal Chemistry: Implications for Drug Design

**DOI:** 10.3390/molecules30143009

**Published:** 2025-07-18

**Authors:** Manuel Novás, Maria J. Matos

**Affiliations:** Departamento de Química Orgánica, Facultad de Farmacia, Universidade de Santiago de Compostela, 15782 Santiago de Compostela, Spain; manuel.novas.gonzalez@rai.usc.es

**Keywords:** trifluoromethyl group, trifluoromethoxy group, drug design, medicinal chemistry

## Abstract

One of the key strategies in drug design involves modifying molecular scaffolds with specific chemical groups, or side chains, to enhance biological and physicochemical properties. These modifications can strengthen interactions with biological targets or improve pharmacokinetic and physicochemical characteristics, factors that are critical in transforming a compound into a viable drug candidate. In this overview, we focus on the presence of trifluoromethyl and trifluoromethoxy groups on different molecules, highlighting their relevance and impact in medicinal chemistry. The discussion and future perspectives in the field are based on a comprehensive review of current literature, with data sourced mainly from SciFinder and PubMed.

## 1. Introduction

In drug design, the structure of a scaffold is critical because it serves as the core framework that determines a molecule’s biological activity, pharmacokinetics, and drug-likeness [1]. The main scaffold provides the foundation for functional groups that interact with biological targets, influencing binding affinity and specificity [2]. A well-designed scaffold ensures optimal orientation of pharmacophores, improving drug efficacy. A rigid scaffold may enhance selectivity but reduce flexibility, affecting bioavailability. A lipophilic scaffold may improve membrane penetration but increase the risk of toxicity [3].

The choice of an ideal scaffold is a fundamental aspect of drug design, influencing everything from target interaction to drug safety and metabolism. However, a well-selected substitution pattern can lead to potent, selective, and safe drugs, making the decoration of a scaffold a crucial consideration in medicinal chemistry. Functional groups determine the chemical reactivity of a molecule under a given set of conditions. Both scaffold and substitution patterns determine properties such as solubility, permeability, and metabolic stability. The amount of possible substitution patterns is very high, with the Craig plot being very helpful for their selection, and an inspiration for medicinal chemists [4].

The presence of fluorine atoms in biologically active molecules can enhance their lipophilicity and, therefore, in vivo uptake and transport [5]. In particular, the trifluoromethyl group (−CF_3_) confers increased stability and lipophilicity in addition to its high electronegativity. In addition, another fluorinated substituent, the trifluoromethoxy group (−OCF_3_), is becoming more and more important in both fields of agrochemicals and pharmaceutical chemistry.

This overview discusses the significance of both functional groups, with a particular emphasis on their role in the design of new drugs. All the information presented in this review was collected from the available literature from online sources. The electronic databases employed for the assortment of relevant information include PubMed, Scifinder, Scopus, Science Direct, Web of Science and Google Scholar. The chemical structures of compounds were drawn using ChemDraw Ultra 23.0.1.11 software. MolCalc^®^ has also been used for calculating electrostatic potential maps, providing insights into electronic distribution and reactivity trends of the compounds containing these functional groups.

## 2. Interest of Fluorine and Methyl-Containing Groups in Drug Design

Fluorine atoms are of great interest in pharmaceutical chemistry due to their small size (1.47 Å), which allows them to replace hydrogen atoms (1.20 Å) in C–H bonds, owing to their steric similarity. Their high lipophilicity combined with their strong electronegativity (4.0 on the Pauling scale), which enables them to act as potent electron-withdrawing groups, helps to explain their widespread use in drug design. Currently, approximately 20% of marketed drugs contain the fluorine atom [6,7].

Also, a significant number of drug candidates in clinical development are halogenated structures [8]. Approximately 40% of lead compounds tested actively contain halogens. Inclusion of halogens on hit compounds is performed in order to exploit steric effects, through the ability of these bulk atoms to occupy the binding site of molecular targets. Fluorination is an excellent strategy due to its high lipophilicity and capacity to increase the absorption of drugs, as said before. The fluorine atom has a unique combination of electronegativity, size, and lipophilicity that contributes to its extensive exploitation in drug discovery. The incorporation of fluorine and fluorinated motifs is a common practice in medicinal chemistry projects with the aim of modulating in vitro potency while controlling relevant ADME-properties (metabolic stability, solubility/lipophilicity balance, bioavailability) at the same time [5].

The first drug incorporating fluorine in its structure was commercialized in 1955. Since then, the presence of fluorine in drugs and agrochemicals has become rather common. Indeed, numerous articles have been published on the importance of fluorine in medicinal chemistry and the many advantages it confers in terms of potency, selectivity, metabolic stability, and solubility, among others [9].

Also, the small, monovalent, and lipophilic methyl group is versatile and of great importance in the design or optimization of bioactive compounds, whether in terms of pharmacodynamic or pharmacokinetic properties [10]. It is known in the academic community as “magic methyl”.

There is an extensive number of articles covering the latest synthetic fluorine incorporation methods, both for the introduction of a single atom and for more complex groups: trifluoromethyl, difluoromethyl, trifluoromethoxy, and many more [11].

## 3. Trifluoromethyl Group

**Figure 1:**
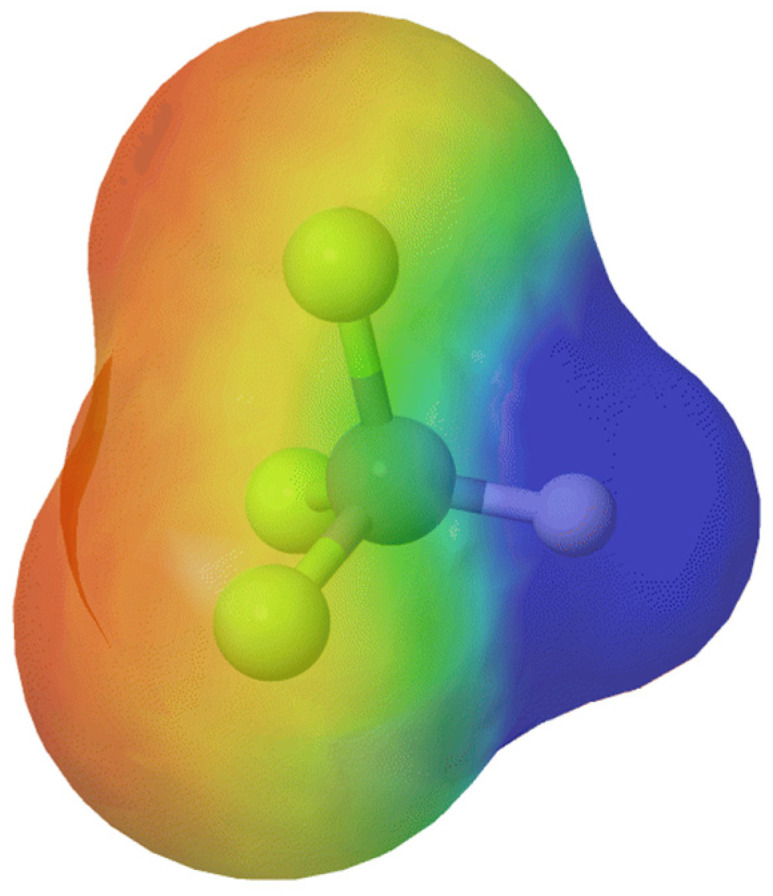
Chemical characteristics of the trifluoromethyl group. Electrostatic potential map in the range of −0.1 to 0.1 obtained using MolCalc^®^, where warm colors indicate higher and blue indicates lower electron density. Blue: positive; red: negative.

In drug discovery, the presence of fluorine in potential drug candidates is a crucial tool in the medicinal chemist’s arsenal. Replacing a hydrogen atom with fluorine to block a metabolic hotspot or adding a trifluoromethyl group to deactivate an aromatic ring are well-established strategies for reducing metabolism, increasing half-life, and lowering drug load. It is important to have in mind the higher electron-withdrawing ability of a trifluoromethyl group compared to a fluorine atom [12]. To better understand the characteristics of this functional group, electrostatic potential maps have been obtained using MolCalc^®^ (Figure 1).

The trifluoromethyl group is one of the most widely used fluorinated moieties in pharmaceuticals. Its presence increases the lipophilicity of molecules, though to a lesser extent than trifluoromethoxy groups, with an Hansch π value of +0.88. The C–F bond is one of the strongest in organic chemistry due to its high polarity, with a bond dissociation energy of 485.3 kJ/mol, compared to 414.2 kJ/mol for a C–H bond. This accounts for the high metabolic stability of the trifluoromethyl group. It is often used as a bioisostere for chlorine atoms due to their steric similarity (1.30 Å vs. 0.99 Å, respectively). The presence of this group has repeatedly been shown to improve solubility, lipophilicity, pharmacokinetic profiles, and binding selectivity of drug candidates [13]. In addition, trifluoromethyl groups can be very stable to chemical, electrochemical, thermal and photochemical degradation [14]. The main drawbacks limiting the broader use of trifluoromethyl groups are the synthetic challenges associated with their incorporation to different scaffolds, including high cost, formation of undesirable byproducts, and difficulties in achieving selectivity [14].

The trifluoromethyl group is also often considered a classical isostere for nonpolar side chains found in proteinogenic α-amino acids, such as methyl (CH_3_), methylthio (SCH_3_), isopropyl (CH(CH_3_)_2_), and ethyl (CH_2_CH_3_) groups. This structural similarity allows for the design of rigid amino acids bearing trifluoromethyl substituents, which can serve effectively as CF_3_-labeled analogs.

The trifluoromethyl group, known for its larger size compared to a methyl group, is among the most frequently used lipophilic substituents in drug design [15]. This group is known to be bulkier than a methyl group. Its presence significantly alters the electronic properties of aromatic rings, a factor that contributes to the effectiveness of several well-known drugs featuring aromatic trifluoromethyl group substitutions in their molecular frameworks. The trifluoromethyl group is a distinctive substituent in organic chemistry, known for its strong electron-withdrawing nature and compact steric profile due to its small van der Waals radius.

The advantages of trifluoromethyl groups over methyl groups are notable and include three specific reasons: (1) Enhanced target binding affinity: The high electronegativity of fluorine atoms makes the trifluoromethyl group a strong electron-withdrawing substituent, which can improve hydrogen bonding and electrostatic interactions with biological targets. Additionally, this group is larger than a methyl group, potentially increasing affinity and selectivity through enhanced hydrophobic interactions. (2) Improved metabolic stability: The strength of the C–F bonds contributes to increased resistance to metabolic degradation, thereby prolonging the drug’s half-life. (3) Modulation of lipophilicity and permeability: The trifluoromethoxy group combines the lipophilicity of the moiety with the polarity of the oxygen atom, allowing fine-tuning of logP values to optimize membrane permeability and bioavailability.

Incorporating a trifluoromethyl group into drug candidates is a significant yet complex strategy in organic and medicinal chemistry. This group is often used to fine-tune physicochemical characteristics and enhance the binding affinity of therapeutic compounds [16]. A comprehensive review on the trifluoromethylation of sp^3^, sp^2^, and sp-hybridized carbons has been presented by Vadim A. Soloshonok and co-authors [16].

Aromatic compounds bearing trifluoromethyl groups are prevalent in pharmaceuticals and advanced organic materials. As a result, significant effort has been directed toward developing efficient methods for introducing trifluoromethyl groups into molecular frameworks. Among these, transition-metal-catalyzed cross-coupling reactions have proven particularly effective for forming C(sp^2^)–CF_3_ bonds. The use of trifluoromethyl copper reagents for aromatic compounds, i.e., as described in references [17,18,19], and other transition metal catalysts (i.e., palladium, nickel) to introduce this group into alkenes, alkynes, and other substrates, i.e., as described in reference [20], are still the most popular approaches. Modification of existing trifluoromethylated compounds, and the use of trifluoromethylating reagents, as Ruppert’s reagent for carbonyl compounds, i.e., as described in references [21,22], are also useful alternatives.

## 4. Trifluoromethoxy Group

**Figure 2:**
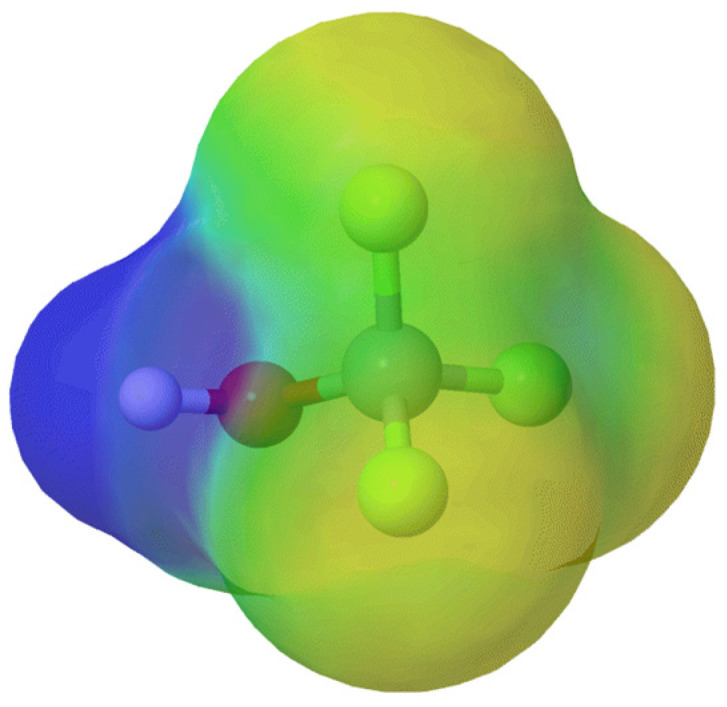
Chemical characteristics of the trifluoromethoxyl group. Electrostatic potential maps in the range of −0.1 to 0.1 obtained using MolCalc^®^, where warm colors indicate higher and blue indicates lower electron density. Blue: positive; red: negative.

Trifluoromethoxy-containing compounds represent a relatively underexplored yet highly promising class of biologically active molecules [23]. Besides five approved drugs, there are also five marketed agrochemicals that contain the trifluoromethoxy group. In fact, this is the most important application of these molecules. The range of therapeutic activities for the drugs available in the market is impressively diverse, as are their scaffolds [16]. To better understand the characteristics of this functional group, electrostatic potential maps have been obtained using MolCalc^®^ (Figure 2).

The trifluoromethoxy substituent offers several metabolic advantages over the methoxy group, largely due to their increased chemical stability and higher electron-withdrawing capacity and lipophilicity [24]. These characteristics make trifluoromethoxy groups more resistant to enzymatic breakdown and less susceptible to forming potentially reactive metabolites. Its greater steric hindrance makes it more difficult for enzymes like CYP450 to access and oxidize the O–C bond, thereby reducing the likelihood of oxidative demethylation [25]. Additionally, the electron-withdrawing effect of the fluorine atoms decreases the electron density on the oxygen atom, making it less susceptible to oxidation [14]. This effect also reduces the ability of the oxygen to act as a hydrogen bond acceptor, which in turn diminishes its interaction with metabolic enzymes [26]. Furthermore, the trifluoromethoxy group is considered one of the most lipophilic substituents, with a Hansch π parameter of +1.04 [27].

The trifluoromethoxy functional group has attracted considerable attention in recent years due to its unique combination of properties, including enhanced metabolic stability, favorable lipophilicity, and distinct electronic characteristics [28]. However, incorporating this group into organic molecules is notoriously challenging, primarily because the trifluoromethoxide anion is highly unstable and difficult to handle directly [29]. Consequently, the pursuit of novel reagents and methodologies for direct trifluoromethoxylation has become an active area of research within the field [30]. Significant advances have been achieved, particularly in the development of nucleophilic and radical trifluoromethoxylating reagents, which have substantially contributed to the expansion of trifluoromethoxy chemistry. There are two main categories related to the chemistry of this functional group: nucleophilic and radical approaches [31]. 

Among the most relevant and widely used methods is the copper-mediated or copper-catalyzed trifluoromethoxylation of aryl halides or diazonium salts. These reactions typically employ silver or cesium trifluoromethoxide (AgOCF_3_ or CsOCF_3_) as a nucleophilic source of the functional group [28]. A classic example is the Sandmeyer-type reaction, in which an aryl diazonium salt reacts with AgOCF_3_ to yield the corresponding aryl trifluoromethyl ether under mild conditions [32]. This method is particularly attractive due to its simplicity and versatility.

More recently, electrophilic and radical-based strategies have expanded the toolbox for the installation of this functional group into different scaffolds. Electrophilic trifluoromethoxylation using hypervalent iodine(III) reagents (i.e., Togni and Umemoto-type reagents) has enabled the direct conversion of phenols or nucleophilic aromatic substrates into trifluoromethoxy-containing molecules [33]. In parallel, visible-light photoredox catalysis has opened new perspectives for trifluoromethoxylation through radical mechanisms, allowing for the incorporation of the functional group under mild and environmentally friendly conditions [34].

## 5. FDA-Approved Drugs Containing Trifluoromethyl and Trifluoromethoxy Groups

Numerous published reviews emphasize the medicinal importance of fluorine-containing compounds as a novel chemical space for the design and development of small molecules, leading to the advancement of both agrochemicals and pharmaceuticals [35]. Analyzing databases that track all drugs reported in phase 3 clinical trials or later stages (though not all may make it to market), it is interesting to observe how many of these molecules contain a fluorine atom in some form, including the functional groups highlighted in this overview. As expected, trifluoromethyl groups are more prevalent than trifluoromethoxy groups due to their favorable properties and better synthetic accessibility.

### 5.1. FDA-Approved Drugs Containing the Trifluoromethyl Group

**Figure 3:**
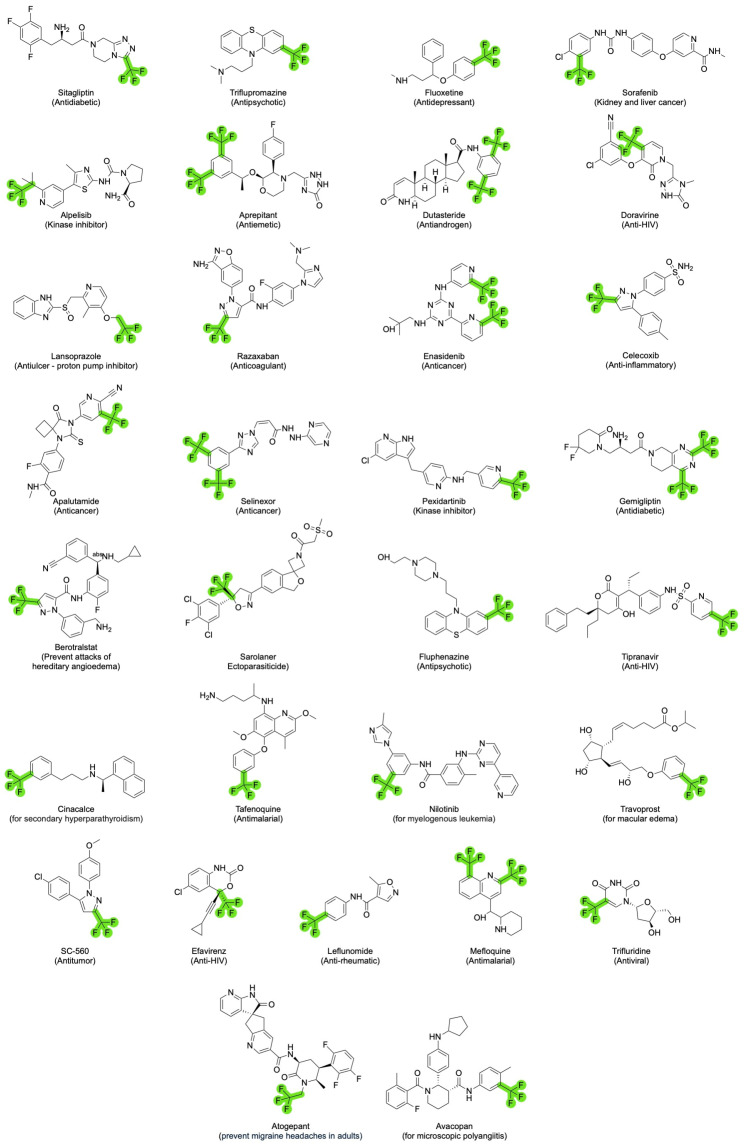
Examples of drugs bearing trifluoromethyl groups (highlighted in green) and their therapeutic applications.

Based on the scope of this overview, the most relevant drugs containing the trifluoromethyl group have been grouped (Figure 3). As observed, there is a wide range of therapeutic applications for the compounds decorated with this versatile functional group. In addition to FDA-approved drugs, several relevant compounds have been investigated in recent decades, including navitoclax (for cancer and myelofibrosis), BAY 38–7271 (with analgesic and neuroprotective properties), and roniciclib (targeting various cancers, including small cell lung carcinoma and solid tumors) [16]. However, most of these molecules were discontinued primarily due to safety concerns.

From the extensive list of drugs approved by the FDA bearing trifluoromethyl groups, a recent molecule can be highlighted: atogepant (Figure 3) [36]. A structure–activity relationship study by Merck led to the development of this compound, a selective oral calcitonin gene-related peptide (CGRP) antagonist. CGRP, a 37-amino acid peptide found in both the central and peripheral nervous systems, is known for its role in processes like vasodilation. Elevated CGRP levels and receptor activity in specific brain areas have been linked to migraine episodes. Therefore, CGRP antagonists can effectively treat acute migraines and inflammatory pain. Approved by the FDA in September 2021, atogepant is used to prevent episodic migraines and cluster headaches [37]. Structurally, atogepant includes two fluorinated components: a trifluorophenyl and a trifluoromethyl group.

Atogepant demonstrates high binding affinity for CGRP receptors in humans and rhesus monkeys, effectively blocking CGRP-induced cAMP signaling in both at low nanomolar concentrations. The drug is selective for CGRP and AMY1 receptors, showing minimal interaction with other receptor types like calcitonin and adrenomedullin. Its IC_50_ values are approximately 0.03 nM for CGRP and 2.40 nM for AMY1 receptors. Dual inhibition may enhance therapeutic outcomes. Additionally, research indicates that atogepant inhibits CGRP-mediated vasodilation in human cranial arteries, particularly in meningeal and cerebral vessels, with less effect in coronary arteries, suggesting that its preventive role in migraines may stem from its action on cranial vasculature. Both trifluoromethyl and trifluorophenyl groups contribute significantly to its pharmacological profile. These fluorinated moieties enhance metabolic stability, improve lipophilicity, and increase binding affinity to the CGRP receptor. Additionally, these groups contribute to better oral bioavailability and blood–brain barrier permeability, which are critical for a drug targeting neurological pathways such as those involved in migraine [37].

### 5.2. FDA-Approved Drugs Containing the Trifluoromethoxy Group

**Figure 4:**
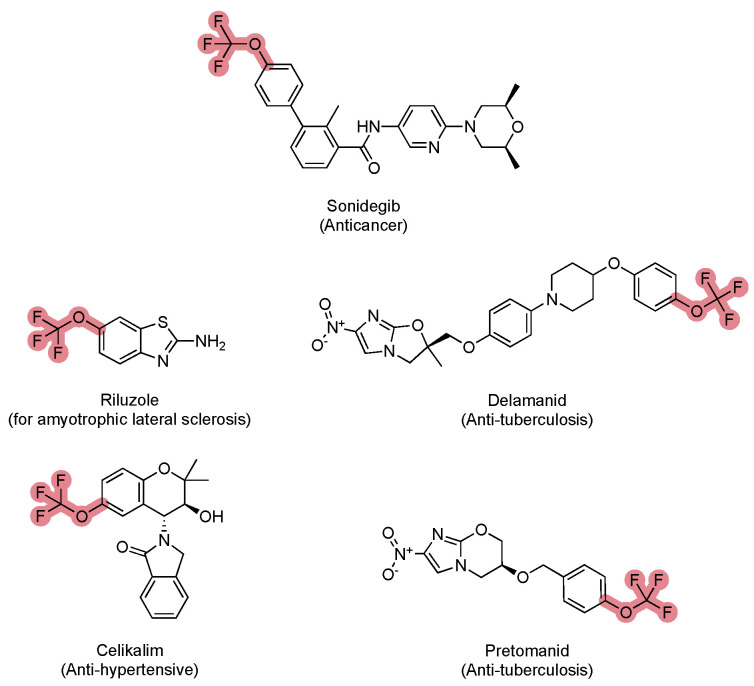
Examples of drugs bearing trifluoromethoxy groups (highlighted in pink) and their therapeutic applications.

There are five known pharmaceuticals containing trifluoromethoxy groups: riluzole (for amyotrophic lateral sclerosis), sonidegib (for cancer), delamanid and pretomanid (for tuberculosis), and celikalim (for blood pressure) (Figure 4). This functional group is much less prevalent in drugs than the trifluoromethyl group. In addition, five marketed agrochemicals are trifluoromethoxy-containing molecules, including the pesticides novaluron and flometoquin, the herbicides flucarbazone-sodium and flurprimidol, and the fungicide thifluzamide. This represents an alternative use for small molecules bearing trifluoromethoxy groups in their structures.

Riluzole stands out as a notable example within this group, as it is one of the few treatment options available for amyotrophic lateral sclerosis, a devastating neurodegenerative disease. The trifluoromethoxy group has been incorporated to enhance the drug’s lipophilicity and membrane permeability, facilitating its passage across the blood–brain barrier. Additionally, this group provides metabolic stability by resisting enzymatic degradation, which improves riluzole’s bioavailability and half-life, ultimately enhancing its pharmacological efficacy in treating neurological conditions.

## 6. Conclusions

Trifluoromethyl and trifluoromethoxy groups play an important role in medicinal chemistry due to their unique physicochemical properties, which deeply influence the biological activity, metabolic stability, and pharmacokinetic profiles of drug candidates. Both groups are present in drugs and drug candidates, with the trifluoromethyl group being significantly more prevalent. Its presence on bioactive molecules can enhance lipophilicity and facilitate membrane permeability, thereby influencing drug–receptor interactions and overall pharmacokinetic behavior. These properties are fundamental requirements to consider when designing new drug candidates. In addition, the trifluoromethyl group’s unique combination of high electronegativity, electron-withdrawing effect, and lipophilicity makes it an excellent alternative to both fluorine atoms and methyl groups. Fluoxetine, a selective serotonin reuptake inhibitor, one of the most commonly prescribed antidepressants, is an excellent example of the impact of the trifluoromethyl group in drug design. The incorporation of this group significantly enhanced the compound’s lipophilicity, thereby improving membrane permeability and facilitating efficient brain penetration. Consequently, trifluoromethyl substitution is widely explored in numerous drug discovery campaigns to optimize potency, selectivity, and metabolic stability. Continued exploration of these groups, including their less-studied derivatives and mixed halogen analogs, promises to expand the toolbox available to medicinal chemists aiming to optimize drug candidates.

## Data Availability

No new data were created or analyzed in this study.
